# Assessment of psychological status by a comprehensive approach in thyroid cancer patients undergoing radionuclide therapy: A feasibility study

**DOI:** 10.1177/00368504241253715

**Published:** 2024-08-08

**Authors:** Alessia Giordano, Ilaria Bochicchio, Rosj Gallicchio, Giovanni Deiana, Rebecca Storto, Anna Nardelli, Michele Di Cosola, Alessandro Lettini, Giovanni Storto

**Affiliations:** 1 Nuclear Medicine, 9267 IRCCS CROB, Referral Cancer Center of Basilicata, Rionero in Vulture, Italy; 2 Psycho-oncology, 9267 IRCCS CROB, Referral Cancer Center of Basilicata, Rionero in Vulture, Italy; 3 18972 Department of Clinical and Experimental Medicine, University of Foggia, Foggia, Italy

**Keywords:** Thyroid cancer, confined hospitalization, hypothyroidism, psychological assessment, and support

## Abstract

This feasibility study evaluated the psychological status of patients with differentiated thyroid cancer (DTC) before, during, and 40 days after administration of I-131 radionuclide therapy (RAI). We investigated the appropriateness of providing patient a comprehensive psychological assessment in an isolation ward. Thirty consecutive patients (Study Group; SG) who received RAI were enrolled. The tools used were the Hospital Anxiety and Depression Scale (HADS) at three different moments, and the Coping Responses Inventory (CRI) at baseline for each patient. A supportive approach was also implemented. Data were collected at the first specialist visit, at the day of admission, and at 40 days follow-up visit. A matched cohort of patients (Control Group; CG), who did not receive psycho-oncological counseling, was retrospectively studied only about their medical needs and requests. Staff exposure to radiation was also compared during SG and CG hospitalization, to assess a possible reduction of radiological risk for them. A significant difference between the basal, intermediate, and final psychological status was observed (p < 0.0001), which was found to be irrespective of the induced hypothyroidism. Patients showed a significant worsening of their status in terms of anxiety and depression after the consent, but it improved 40 days after treatment. Repeated measures analysis showed a similar trend in patients’ psychological status over this period. At hospital discharge, patients showed indirect signs of increased well-being. CG required more nursing and medical interventions. Staff exposure was significantly lower during hospitalization of SG as compared to CG. This study demonstrates that timed psychological evaluation and appropriate support may help to reduce anxiety and depression of patients receiving a diagnosis of cancer and undergoing RAI. Moreover, an improvement of workplace safety was recorded.

## Introduction

Management of patients with differentiated thyroid cancer (DTC) undergoing radionuclide therapy represents a challenge due to both historical misconceptions and recent legal limits.^1–4^ Patients candidate for admission to nuclear medicine isolation rooms may need timely psychological assessment and support before, during, and after hospitalization. Nevertheless, the most appropriate moment for psychological assistance has not been widely confirmed by standard operative procedures and remains uncertain in such setting. At present, psychological support is not routinely provided to these patients and their emotional needs are underestimated. I-131 radionuclide therapy (RAI) preparation requires several preliminary operations in charge of a multidisciplinary staff, but it does not still consider psychological support as essential at this stage.^5^ Patients must undergo hypothyroidism by l-thyroxine withdrawal or TSH exogen stimulation (rhTSH), and a hypo-iodine diet is also implemented. Psychological support is provided only upon request. There is clinical evidence for patients, already subjected to surgery, coping with hypothyroidism that in the proximity of RAI and isolation their psychological status is not optimal and frequently leads to misapprehension, anxiety, and depression mood.^6,7^ The value of psycho-oncology is well known.^8^ Among the tools to evaluate the quality of life, the Hospital Anxiety and Depression Scale (HADS) and the Edmonton Symptom Assessment System (ESAS) represent validated procedures using standard questionnaires.^9–11^ The Coping Responses Inventory (CRI) can be used as an additional aid to assess problem solving through different types of coping strategies.^12,13^ This methodology has already been implemented successfully in patients withdrawing their l-thyroxine intake, hence experiencing hypothyroidism,^14^ but was never tested on patients undergoing rhTSH administration. Achievement of the abovementioned multidisciplinary approach for this setting (including psycho-oncological assessment) will reduce patients’ discomfort and will improve their well-being despite stressful circumstances. Accordingly, a decreased demand for patients’ direct care during isolation can be expected. Reduced professionals’ radiation exposure will be in line with current guidelines regarding job and environmental safety.^15^ Overall, multispecialty patient management constitutes a suitable model. This study aimed to investigate whether psycho-oncological evaluation and support gave any benefit to patients with previous DTC (with or without—exogen-induced hypothyroidism) suffering of coping following RAI and isolation. Concurrently, we aimed to verify if psycho-oncological support also reduced the patient's needs for direct care soon after RAI as compared with the control group (CG) not receiving assistance, and whether psycho-oncological support improved the management of patients undergoing radioactive iodine therapy with a consequent mitigation of radiation exposure risk for staff.

## Materials and methods

### Patients

This study enrolled 30 consecutive patients (12 men and 18 women; mean age, 51 ± 18 years) from April to June 2022 at IRCCS CROB, Referral Cancer Center of Basilicata, Italy. All patients underwent RAI and were admitted to an isolation ward of nuclear medicine for a standard duration of three days after radiation. All of them received a psycho-oncology assessment and support, before, during, and after hospitalization. Inclusion criteria were age above 18 years; pregnancy test negative; total or near-total thyroidectomy 2 to 6 months before RAI; histology with papillary or follicular histotype, surgical stage ≥ pT1; induced hypothyroidism; scheduled hospitalization in an isolation ward. Patients were treated with an empirical radioiodine activity ranged from 3700 MBq to 4440 MBq, according to tumor histological type and staging. Metastatic disease, previous serious medical conditions, prior or current psychiatric illness, and habitual use of psychotropic substances were considered exclusion criteria. Patients with matching clinicopathological features (number = 30, female to male ratio, type of tumor, eligibility for iodine therapy) hospitalized in isolation constituted the CG and were retrospectively studied only concerning their medical needs and requests. The CG served to assess the frequency and type of daily demands of patients hospitalized in isolation as compared to that of the study group, retrieving patients’ data from medical records. They were evaluated considering the number, type, and timing (day/night) of complaints during the hospitalization, irrespective of their nature (e.g. from referred headache to the compulsory need for coffee). They not received psycho-oncological counseling.

Our institutional review board approved this study (protocol # 20210009766 / CEUR 35/2020). All patients who underwent RAI together with psycho-oncology assessment signed an informed consent form in accordance with the Declaration of Helsinki. They had the decisional capacity to provide written informed consent themselves.

### Radioiodine treatment

Patients of the study group were separated into two groups based on the hypothyroidism induction approach: fifteen patients discontinued Levothyroxine replacement therapy, 1 month before hospitalization; the other group (#15), underwent exogenous TSH stimulation with recombinant human TSH (rhTSH). All patients had to follow a diet without iodine 2 weeks before RAI. Patients were admitted after testing negative for SARS-CoV-2. On the first day, patients received oral administration of a tailored activity of I-131 and after 3 days in isolation were discharged to home with a general behavioral guide according to current radiation safety rules. Levothyroxine therapy was gradually resumed for patients who discontinued the treatment.

### Assessment of psychological status

All patients enrolled received two standard questionnaires: HADS to evaluate anxiety and depression, CRI for coping mechanisms. Self-assessment scale results of HADS were expressed for both anxiety and depression. This is a 14-questions survey designed to assess depression and anxiety in the setting of a hospital ward. The depression items tend to focus on the anhedonic symptoms of depression. The anxiety and depressive subscales are also suitable to measure the severity of the emotional disorder. Each question is rated from 0 to 3 on a 4-point severity scale. The HADS produces two scales, one for anxiety (HADS-A) and one for depression (HADS-D), differentiating the two states. Scores greater than or equal to 11 on either scale indicate a definitive case. CRI was used at baseline encompassing two main conceptual domains: cognitive approach strategies and cognitive avoidance strategies. Both domains included four items for each strategy: logical reasoning, positive reevaluation, behavioral approach strategies, problem solving, cognitive avoidance, acceptance or resignation, behavioral avoidance strategies, and emotional outbursts. On a patient basis, the number of normal, borderline, or pathological approaches was reported for each domain. They were also guided in completing the questionnaires. Moreover, patients joined unscheduled psychological interview according to their psychological status. The semi-structured psychological interview is a widely used data collection technique in psychology characterized by a combination of predetermined questions tracing the psychic and intrapsychic functioning of the patient. This approach allows flexibility to the researcher, which can ask tailored questions based on the participant's responses, hence adapting the support. The combination of defined and open-ended questions makes it possible to balance data standardization with the ability to explore inner experiences. The semi-structured interview is a valuable tool for research purposes. Generally, the questions were aimed at investigating self-coherence, reality examination, interpersonal relationships, and defense and rigidity.

Daily needs and demands of patients were routinely registered during the hospital permanence. Particularly, the psychological assessment was carried out at three different moments: the days (2–7) before hospitalization (Time 0), during pre-hospitalization procedures (as per pandemic-related issues and medical preliminary assessment); at the time of admission, for administration of radioactive iodine (Time 1), and 1 month after discharge (Time 2). The visits were replicable, although performed with a different methodology in reason of radioprotection control. At first approach, patients underwent a direct assessment whereas phone interviews were performed afterward. Questionnaires at Time 1 were completed by patients but, the psychological support was warranted by a phone interview to avoid prolonged and close contact with radioactive patients. At the follow-up assessment (Time 2) patients went through phone interview and counseling, and the questionnaires were completed by the psycho-oncologist.

### Workplace safety assessment

All nurses involved in the radiometabolic ward (eight on the roster) were monitored for the radiation dose during the trial. The mean dose rates were measured by thermoluminescent dosimeters (TLDs) used to record the radiation dose (gray/Gy) accumulated by the staff. TLDs do not yield immediate information about radiation exposure and were monthly checked retrospectively. The data of the same professionals were compared with those registered during hospitalization of the CG.

## Statistical analysis

Continuous data were expressed as percentages, median, and mean ± SD as appropriate. Comparison between the mean values was performed with paired Student's *t*-test (two-tailed probability), when necessary. One-way analysis of variance (one-way ANOVA test) with Sheffè's method (post-hoc test in ANOVA) was used to test the difference between the means of subgroups of variables. To compare data under different conditions, we used the repeated measures analysis of variances (within-subjects effect). Our hypotheses were tested using Bonferroni-adjusted alpha levels. The Greenhouse–Geisser and Huynh–Feldt corrections^16,17^ attempt to estimate epsilon (sphericity). Sphericity refers to the similarity of variances of differences between measurements. With this method, an epsilon of 1 indicates that the condition of sphericity is exactly met. As epsilon decreases below one, the greater is the violation of sphericity. Moreover, the Friedman test was performed to assess the difference between several related measures. Correlation analysis (*correlation coefficient r*) was used to determine whether the values of variables were associated, when requested. A *p* value <0.05 was considered statistically significant.

## Results

**Figure 1.**
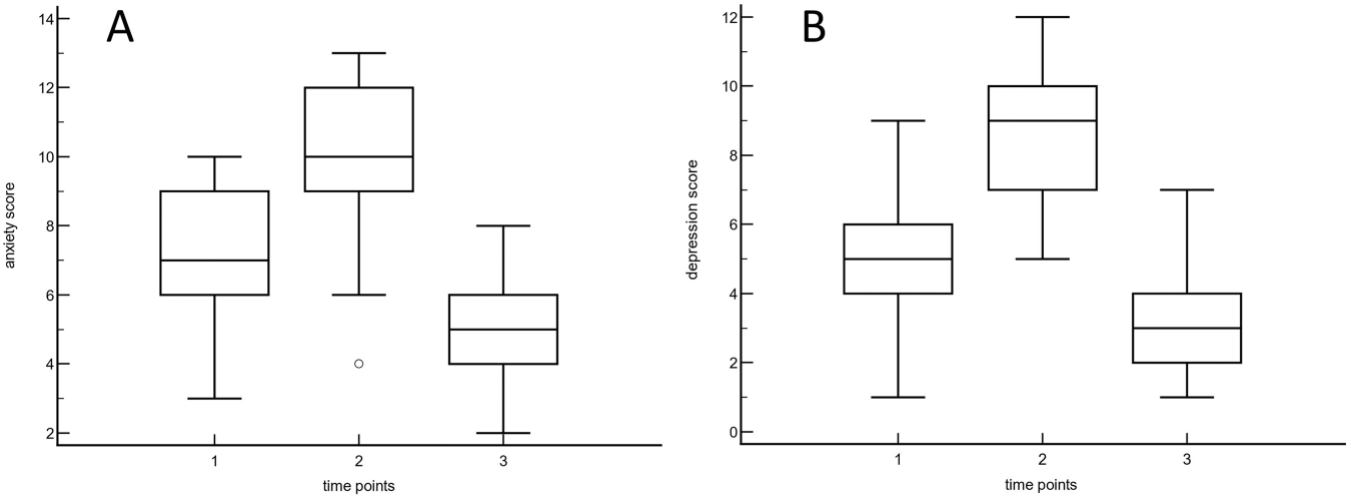
Box-and-whisker plots of HADS scores concerning anxiety (A) and depression (B), before (1), during (2), and after (3) hospitalization.

**Figure 2.**
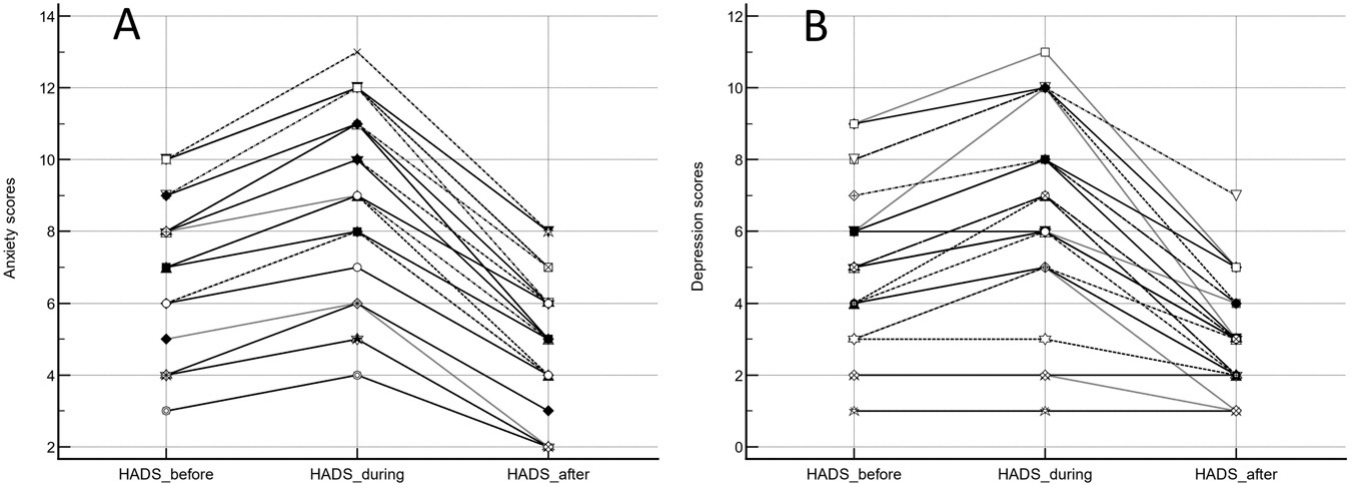
Dot and line HADS diagram on individual basis concerning anxiety (A) and depression (B) before, during and after hospitalization.

**Figure 3.**
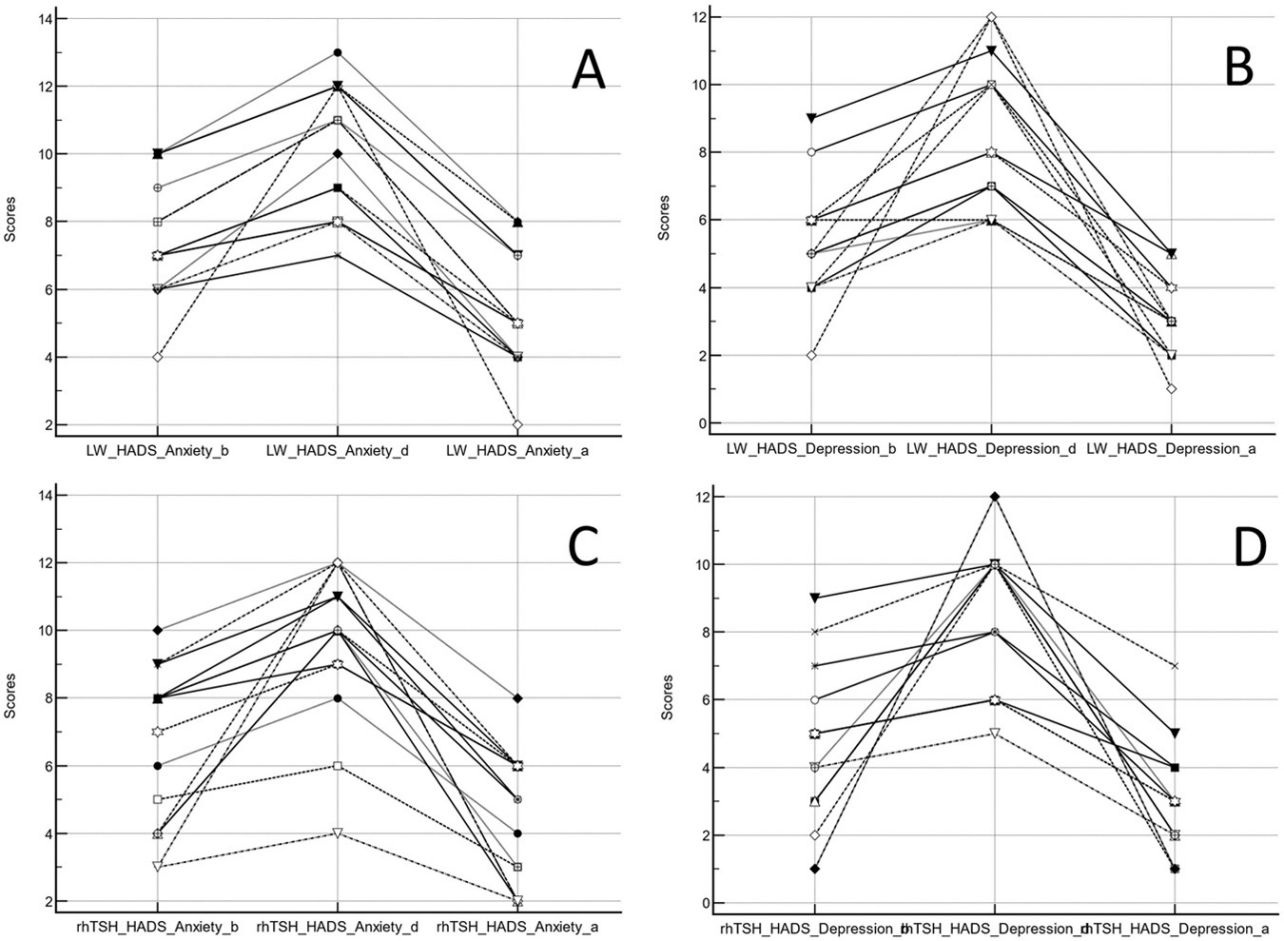
Diagrams of HADS scores for anxiety (A, C) and depression (B, D) in patients who withdrew Levothyroxine (LW) and those who received recombinant thyrotropin (rhTSH), before (b), during (d) and after (a) hospitalization.

Overall patients’ characteristics are shown in Table 1. In the study group, the patients who underwent rhTSH stimulation presented higher TSH values at the zenith, as normally encountered in daily routine. The mean HADS over time and baseline CRI results for each domain are presented in Table 2, while overall HADS box-and-whisker plots are reported in Figure 1. Figure 2 shows scores obtained on an individual basis. Diagrams with HADS scores concerning patients who withdrew Levothyroxine and those who received rhTSH are shown in Figure 3. Differences between scores at each time point were statistically significant (*p* < 0.0001; Table 3). Data were reported for both anxiety and depression, with increasing values from Time 0 to Time 1, reaching minimum levels 1 month later. No statistically significant difference over time was observed between the group of patients who withdrew Levothyroxine and those who did not.

**Table 1.** Overall individual data of DTC patients.

Characteristics	Study group	Control group
**Total number of patients**	30	30
**Age at diagnosis, year, median (range)**	51 (33–69)	50 (29–70)
**Sex F/M**	18/12	20/10
**Total Thyroidectomy Histology**	30 (100)	30 (100)
Papillary (%)	22 (73)	25 (84)
Follicular (%)	5 (17)	2 (6)
Variants* (%)	3 (10)	3 (10)
**Tumor max diameter, median (range) pT (TNM), number**	1.55 (0.6–5.2)	2.1 (0.4–5.0)
pT1	18	15
PT2	10	6
PT3	2	9
**TSH µUI/ml, median (range)**		
rhTSH group	153 (62–204)	131 (66–250)
withdrawn group	89 (49–189)	91 (32–201)
**HTg ng/ml, median (range)**		
rhTSH group	1.39 (0.07–14.2)	2.0 (0.2–5.8)
withdrawn group	1.0 (0.1–116)	1.7 (0.1–19.9)

DTC, differentiated thyroid cancer; *, hurtle, tall cell, columnar, sclerosing; TNM, tumor, node, metastasis stage; TSH, thyroid-stimulating hormone; rhTSH, recombinant human thyrotropin; HTg, human serum thyroglobulin.

**Table 2.** Mean values of Hospital Anxiety and Depression Scales at different time points and Coping Responses Inventory at baseline.

Category	Mean ± SD			
**HADS anxiety_b_**	7.0 ± 2.2			
**HADS anxiety_d_**	9.9 ± 2.1			
**HADS anxiety_a_**	4.9 ± 1.9			
**HADS depression_b_**	4.9 ± 2.1			
**HADS depression_d_**	8.7 ± 2.2			
**HADS depression_a_**	2.9 ± 1.4			
**CRI at baseline (number)**		**Normal**	**Borderline**	**Pathological**
Cognitive Approach Strategies		43	45	32
Cognitive Avoidance Strategies		50	50	20

HDSA, Hospital Anxiety and Depression Scale; b, before hospitalization; d, during hospitalization; a, after hospitalization; CRI, Coping Responses Inventory.

**Table 3.** Pairwise comparisons of HADS values before, during and after hospitalization for both anxiety and depression.

Factors			Mean difference	Std. Error	*p* ^a^	95% CI^a^
HADS Anxiety before	–	HADS Anxiety during	−2933	0404	<0,0001	−3959 to −1907
HADS Anxiety before	–	HADS Anxiety after	2167	0118	<0,0001	1866 to 2467
HADS Anxiety during	–	HADS Anxiety after	5100	0391	<0,0001	4108 to 6092

**Table:** 

Factors			Mean difference	Std. Error	*p* ^a^	95% CI^a^
HADS Depression before	–	HADS Depression during	−3800	0600	<0,0001	−5324 to −2276
HADS Depression before	–	HADS Depression after	1933	0209	<0,0001	1403 to 2463
HADS Depression during	–	HADS Depression after	5733	0493	<0,0001	4479 to 6987

a Bonferroni corrected; HDSA, Hospital Anxiety and Depression Scale.

Concerning the approach coping mechanism 35.8% of responses were normal, 37.5% borderline, and 26.7% pathological, whereas 41.7% of avoidance coping mechanisms were normal, 41.7% borderline, and 16.6% pathological. At last, approximately 61% (58–64%) of items showed a non-adaptive type of response to stress. At correlation analysis no significant relationship was found between patients’ age and both HADS and CRI scores throughout the time (*r* range: 0.003–0.5; *all p*: ns).

Moreover, from the analysis of medical records, the matched CG of patients not subjected to psycho-oncology support required more nursing and medical interventions. On a patient basis, overall needs during hospitalization were 4 in the SG and 23 in the CG (*p* < 0.0001). Two patients referred transient headache in SG and 12 in CG (*p* < 0.05). One patient required intervention for stomach spasms in SG and 10 in the CG (*p* < 0.01). All complaints registered in medical records were likely linked to anxiety and stress because of their rapid reversibility. One patient in each groups experienced a transient swollen tender neck (radioiodine-related).

The monthly mean absorbed dose rates for the 8 staff unit exposed to radioactive patients were 0.7 ± 0.2 mSv *versus* 1.2 ± 0.5mSv, the latter referring to consolidated historical data recorded during CG hospitalization (*p* < 0.001).

## Discussion

There are several issues arising during management of patients undergoing radionuclide therapy and that have to be hospitalized in an isolated, controlled ward.^18^ The psychological status of subjects represents a critical concern. In fact, they are generally hypothyroid, they are conscious of having had cancer, become aware of being isolated and receiving radioactive drugs. It is also important to consider the public misconception surrounding this therapy due to recent accidents and environmental radioactive events.^19^ As stated above, most patients belonging to this setting exhibit a mishmash of distress conditions. It is well known that patients referred to a nuclear medicine ward for therapy have several anxiety-predisposition factors (e.g. hypothyroidism, cancer-related apprehension, isolation admission distress).^20^ Our study was designed to establish whether a sequential psycho-oncological approach before, during, and after admission of patients undergoing RAI can be implemented and if this setting could benefit from a sustained comprehensive timely support. A better radioactive patient management was also expected. The results showed that the patient's distress (anxiety and depression) was mitigated during pre-hospitalization days, it worsened at the time of radioactive drug administration and significantly decreased 1 month later. This finding, although not completely new^14^ was obtained for the first time in all patients’ categories undergoing RAI, those presenting endogen hypothyroidism, and patients subjected to preparation with rhTSH administration. There were no differences between the two groups. It can be doubted that the pre-hospitalization days are near to admission and consequently the psychological status of patients is sensibly similar. However, during the preparation procedures, apart from psychological support, patients were enrolled into a familiar environment with the support of staff; the nuclear medicine doctor is committed to explaining to them the benefits, adverse reactions, and complications of the treatment. In patients with DTC ablation success or diagnostic uptake requires high TSH stimulation to reach target levels. Unfortunately, this condition is accompanied by symptoms such as fatigue, weight gain, constipation, cold intolerance, coarse skin and hair, hair loss, decreased memory, muscle aches, weakness, and depression. It encompasses a psychological status that should be recognized and that is usually worsened in a context of general misapprehension. Patients undergoing RAI are conscious of their cancer, even after surgery, and they are worried about radiometabolic therapy and isolation. This distressed scenario needs the development of the best attainable supportive care system involving professionals such as nurses, nuclear medicine doctors, technical staff, and psycho-oncologists. Nevertheless, Banihashem et al.^14^ found that in a cohort of patients with previous DTC undergoing l-thyroxine withdrawal and radioiodine, HADS scores physiologically lowered over 6 months after RAI, indicating a gradual improvement of quality of life and no need for psychological follow-up.^14^ These data do not appear fully coherent with ours. On the other hand, Javaloyes et al.^21^ reported that in a non-randomized controlled study, hypothyroid patients who received a psycho-oncological intervention based on counseling showed a statistically significant decrease in anxiety and depression, registered from baseline to post-treatment period, compared to a similar group who did not.^21^

In our study patients were evaluated for their distress but they also received unplanned psychological support. Anxiety and depression were significant at the first visit, reaching the highest level at the day of treatment. These data support the hypothesis that a timely and real help could be provided on the day of radionuclide administration, or that maximum care engagement is required at that moment. It is reasonable that the first psychological approach (the days before hospitalization) was partially inefficient or insufficient considering the worsening of patients’ moods. However, after the drug administration, we detected initial signals of improvement, thereafter, confirmed at the 1-month evaluation. Patients who attended the first two psychological visits were less demanding for the following 2 days after admission compared to those of CG. It can be speculated that once received the radioactive therapy they can become relaxed, though the beneficial effect of psychological support cannot be excluded. Our patients were helped with both interview at admission and targeted psychological intervention. In fact, the interview was used as a way to support them through this experience of awareness of illness as well as to compensate for their distress, according to other authors.^22–25^ Like others, we detected significant psychological stress that justified the support interventions.^26–28^ In the context of the abovementioned misapprehension, anxiety, and depression, patients frequently will ask for staff support. Unfortunately, this occurrence can increase the exposure to radiation of the professional staff. In addition, within the environment of the radiometabolic ward, it is mandatory to respect the dose limits that various groups may receive, including staff and the public. Iodine-131 results in the largest dose to medical staff, caregivers, and relatives.^29^ Providing patients at hospitalization with psycho-oncological support would improve management also in terms of workplace safety, avoiding and reducing unnecessary exposure. Patients who received the psycho-oncologist aid required less, and often banal, staff support. The feasibility of this approach justified the need and the timing of the psycho-oncological support provided to our patients. It seemed that psychological support on the day of admission is not an important benefit, for this reason the procedure might be started on the subsequent day. This model could be easily implemented in daily practice. Even though hypothyroidism seems to be a dominant factor in this contest, our study demonstrated that patients with l-thyroxine withdrawal, in preparation for RAI, suffered isolation similarly to patients who underwent exogenous stimulation with rhTSH. From a pathophysiological point of view, it could be suggested that the effects of rhTSH at zenith time may reveal its efficacy but also the associated sequence of side effects. In fact, clinical studies show that patients experiencing acute hypothyroidism suffered greater levels of anxiety and depression as well as lesser control of motor activities.^30–32^

It appears that isolated patients could be managed easily in terms of both professionals’ safety and patients’ control. There is evidence for implementing a comprehensive approach to patients, including psycho-oncology assessment. From an organizational point of view, our data sustain the hypothesis of improving multidisciplinary patient management hospitalized in isolation rooms because of their radioactivity.^33^ Repeated measures analysis confirmed the mutability of psychological status over time, hopefully finding the optimal moment to implement supportive care.

### Clinical implications

This study demonstrates that timely and appropriate psychological evaluation and tailored support on specific needs may be valuable for patients undergoing radionuclide therapy with I-131 for thyroid cancer.

### Study limitations

The results of our work should be interpreted in the view of certain limitations. First, this study included patients that strictly fulfilled inclusion criteria but was a monocentric study with a relatively small cohort. Moreover, the wide age range of patients was found not correlated with the psychological reaction, and the impact of all other intrinsic variables, if any, has not been figured out.

### Conclusion

This study shows that the implementation of a psycho-oncological evaluation and support of patients hospitalized for radionuclide therapy in an isolation ward is feasible within the logistic limits. It could be useful to spend more time on patients’ needs to reassure them, this will also reflect on workplace management optimization.

## Supplemental Material

sj-pdf-1-sci-10.1177_00368504241253715 - Supplemental material for Assessment of psychological status by a comprehensive approach in thyroid cancer patients undergoing radionuclide therapy: A feasibility study Supplemental material, sj-pdf-1-sci-10.1177_00368504241253715 for Assessment of psychological status by a comprehensive approach in thyroid cancer patients undergoing radionuclide therapy: A feasibility study by Alessia Giordano, Ilaria Bochicchio, Rosj Gallicchio, Giovanni Deiana, Rebecca Storto, Anna Nardelli, Michele Di Cosola, Alessandro Lettini and Giovanni Storto in Science Progress
