# Supplementary material for: Assessment of psychological status by a comprehensive approach in thyroid cancer patients undergoing radionuclide therapy: A feasibility study
Source: Sci Prog. 2024 Aug 8;107(3):00368504241253715. doi: 10.1177/00368504241253715 (PMC11318648; doi:10.1177/00368504241253715)
Supplement: sj-pdf-1-sci-10.1177_00368504241253715 - Supplemental material for Assessment of psychological status by a comprehensive approach in thyroid cancer patients undergoing radionuclide therapy: A feasibility study [file sj-pdf-1-sci-10.1177_00368504241253715.pdf]

Hospital Anxiety and Depression Scale (HADS)

Tick the box beside the reply that is closest to how you have been feeling in the past week.
Don’t take too long over you replies: your immediate is best.

D	A		D	A	
		**I feel tense or 'wound up':**			**I feel as if I am slowed down:**
	3	Most of the time	3		Nearly all the time
	2	A lot of the time	2		Very often
	1	From time to time, occasionally	1		Sometimes
	0	Not at all	0		Not at all
		**I still enjoy the things I used to enjoy:**			**I get a sort of frightened feeling like 'butterflies' in the stomach:**
0		Definitely as much		0	Not at all
1		Not quite so much		1	Occasionally
2		Only a little		2	Quite Often
3		Hardly at all		3	Very Often
		**I get a sort of frightened feeling as if something awful is about to happen:**			**I have lost interest in my appearance:**
	3	Very definitely and quite badly	3		Definitely
	2	Yes, but not too badly	2		I don't take as much care as I should
	1	A little, but it doesn't worry me	1		I may not take quite as much care
	0	Not at all	0		I take just as much care as ever
		**I can laugh and see the funny side of things:**			**I feel restless as I have to be on the move:**
0		As much as I always could		3	Very much indeed
1		Not quite so much now		2	Quite a lot
2		Definitely not so much now		1	Not very much
3		Not at all		0	Not at all
		**Worrying thoughts go through my mind:**			**I look forward with enjoyment to things:**
	3	A great deal of the time	0		As much as I ever did
	2	A lot of the time	1		Rather less than I used to
	1	From time to time, but not too often	2		Definitely less than I used to
	0	Only occasionally	3		Hardly at all
		**I feel cheerful:**			**I get sudden feelings of panic:**
3		Not at all		3	Very often indeed
2		Not often		2	Quite often
1		Sometimes		1	Not very often
0		Most of the time		0	Not at all
		**I can sit at ease and feel relaxed:**			**I can enjoy a good book or radio or TV program:**
	0	Definitely	0		Often
	1	Usually	1		Sometimes
	2	Not Often	2		Not often
	3	Not at all	3		Very seldom

Please check you have answered all the questions

Scoring:

Total score: Depression (D) ___________ Anxiety (A) ______________

0 -7 = Normal

8 -10 = Borderline abnormal (borderline case)

1 1-21 = Abnormal (case)

Zigmond AS, Snaith RP. The Hospital Anxiety and Depression Scale. Acta Psychiatrica Scandinavica 1983; 67: 361e370.

Coping Responses Inventory

Part 1

You should think about the most important problem or the most stressful situation that has happened to you in the last 12 months (see: issues with relatives or friends, an illness or death of a relative, an accident, economic or workplace problem). Please briefly describe the problem below.

Now answer each of the following 10 questions below about the abovementioned situation

DN Decisively NO

PN Mainly NO

PY Mainly Yes

SY Decisively Yes

	DN	PN	PY	SY
1. Have you ever faced a problem like this before?	☐	☐	☐	☐
2. Did you know this problem would happen to you?	☐	☐	☐	☐
3. Have you had enough time to prepare to handle this problem?	☐	☐	☐	☐
4. When the problem occurred did you consider it as a threat?	☐	☐	☐	☐
5. When the problem occurred did you see it as a challenge?	☐	☐	☐	☐
6. Was the problem caused by something you did?	☐	☐	☐	☐
7. Was the problem caused by something someone else did?	☐	☐	☐	☐
8. Has anything good come out from addressing this problem?	☐	☐	☐	☐
9. Has this problem been resolved?	☐	☐	☐	☐
10. If the problem solved, is everything fine for you?	☐	☐	☐	☐

Part 2

Carefully read each of the following 48 questions and indicate how often you performed that behavior in relation to the problem you described in part 1.

Mark:

N Never

R Rarely

S Sometimes

O Often

		N	R	S	O
1.	Have you thought about several ways to address the problem?	☐	☐	☐	☐
2.	Have you said things to yourself to make you feel better?	☐	☐	☐	☐
3.	Have you talked about this problem with your partner or relatives?	☐	☐	☐	☐
4.	Have you prepared an action plan and followed it?	☐	☐	☐	☐
5.	Have you tried to forget the whole thing?	☐	☐	☐	☐
6.	Did you have the feeling that time would make a difference?	☐	☐	☐	☐
7.	Have you tried to help other people facing a similar problem?	☐	☐	☐	☐
8.	Did you take it out on other people when you felt depressed or angry?	☐	☐	☐	☐
9.	Have you tried to take a step back from the situation?	☐	☐	☐	☐
10.	Have you reminded yourself how much worse the things could be?	☐	☐	☐	☐
11.	Have you talked about the problem with a friend?	☐	☐	☐	☐
12.	Did you know what needed to be done better?	☐	☐	☐	☐
13.	Have you tried not to think about the issue?	☐	☐	☐	☐
14.	Did you realize that you have not control over the problem?	☐	☐	☐	☐
15.	Have you engaged in new activities?	☐	☐	☐	☐
16.	Did you take a risk and do something risky?	☐	☐	☐	☐
17.	Have repeated in your mind what you would say or do?	☐	☐	☐	☐
18.	Have you tried to see the positive side?	☐	☐	☐	☐
19.	Have you spoken to an expert (e.g. doc)?	☐	☐	☐	☐
20.	Have you decided what you wanted?	☐	☐	☐	☐
21.	Did you daydream a better time/place?	☐	☐	☐	☐
22.	Did you think that the outcome would be decided by fate?	☐	☐	☐	☐
23.	Have you tried to make new friends?	☐	☐	☐	☐
24.	Have you kept yourself distant from others?	☐	☐	☐	☐
25.	Did you try to anticipate how things would end?	☐	☐	☐	☐
26.	Did you think how better you was than others?	☐	☐	☐	☐
27.	Did you search for help from other similar people?	☐	☐	☐	☐
28.	Have you tried at least to different solving ways?	☐	☐	☐	☐

29. Did you try to delay of thinking the problem? ☐ ☐ ☐ ☐
30. Did you accept the situation? ☐ ☐ ☐ ☐
31. Have you read more than usual? ☐ ☐ ☐ ☐
32. Did you shout to vent? ☐ ☐ ☐ ☐
33. Have you tried to find some personal meaning in the situation? ☐ ☐ ☐ ☐
34. Have you tried telling yourself that things would be better? ☐ ☐ ☐ ☐
35. Have you tried to find out more about the situation? ☐ ☐ ☐ ☐
36. Have you tried to learn to do more on your own? ☐ ☐ ☐ ☐
37. Have you wished the problem would go away? ☐ ☐ ☐ ☐
38. Did you expect the worst possible outcome? ☐ ☐ ☐ ☐
39. Have you spent more time on funny activities? ☐ ☐ ☐ ☐
40. Have you cried to express your feelings? ☐ ☐ ☐ ☐
41. Did you try to anticipate the new ongoing questions? ☐ ☐ ☐ ☐
42. Have you thought about how this event positively change your life? ☐ ☐ ☐ ☐
43. Have you prayed for guidance or strength? ☐ ☐ ☐ ☐
44. Have you coped the issue step by step? ☐ ☐ ☐ ☐
45. Did you try to deny how serious the problem really was? ☐ ☐ ☐ ☐
46. Have you lost hope? ☐ ☐ ☐ ☐
47. Have you turned to work to help yourself? ☐ ☐ ☐ ☐
48. Did you do something you didn’t think would work? ☐ ☐ ☐ ☐
